# Maxillary molar distalization with aligners in adult patients: a multicenter retrospective study

**DOI:** 10.1186/s40510-016-0126-0

**Published:** 2016-04-18

**Authors:** Serena Ravera, Tommaso Castroflorio, Francesco Garino, Sam Daher, Giovanni Cugliari, Andrea Deregibus

**Affiliations:** Post-Graduate School of Orthodontics, Lingotto – Dental School, Department of Surgical Sciences, University of Turin, Turin, Italy; Private practice, Turin, Italy; Department of Orthodontics, Dugoni School of Dentistry, University of the Pacific, San Francisco, CA USA; Department of Statistics and Quantitative Methods, University of Milano-Bicocca, Milan, Italy

**Keywords:** Class II, Aligners, Molar distalization, Adult patients

## Abstract

**Background:**

The aim of the present study was to test the hypothesis that bodily maxillary molar distalization was not achievable in aligner orthodontics.

**Methods:**

Forty lateral cephalograms obtained from 20 non-growing subjects (9 male, 11 female; average age 29.73 years) (group S), who underwent bilateral distalization of their maxillary dentition with Invisalign aligners (Align Technology, Inc., San José, CA, USA), were considered for the study. Skeletal class I or class II malocclusion and a bilateral end-to-end class II molar relationship were the main inclusion criteria. Cephalograms were taken at two time points: (T0) pretreatment and (T2) post-treatment. Treatment changes were evaluated between the time points using 39 variables by means of paired *t* test. The level of significance was set at *P* < 0.05.

Reproducibility of measurements was assessed by the intraclass correlation coefficient (ICC).

**Results:**

The mean treatment time was 24.3 ± 4.2 months. At the post-treatment point, the first molar moved distally 2.25 mm without significant tipping (*P* = 0.27) and vertical movements (*P* = 0.43). The second molar distalization was 2.52 mm without significant tipping (*P* = 0.056) and vertical movements (*P* = 0.25). No significant movements were detected on the lower arch. SN^GoGn and SPP^GoGn angles showed no significant differences between pre- and post-treatment cephalograms (*P* = 0.22 and *P* = 0.85, respectively).

**Conclusions:**

Aligner therapy in association with composite attachments and class II elastics can distalize maxillary first molars by 2.25 mm without significant tipping and vertical movements of the crown. No changes to the facial height were revealed.

## Background

The distalization of maxillary molars is frequently required in class II non-extraction patients. Resolving class II molar relationships by distalizing maxillary molars may be indicated for patients with minor skeletal discrepancies [1].

The upper molars can be distalized by means of extra or intraoral forces [2]. In recent years, several techniques have been developed to reduce the dependence on patient compliance, such as intraoral appliances with and without skeletal anchorage. However, even these devices can produce undesirable tipping of the maxillary molars and/or loss of anterior anchorage during distalization [3–9]. In the last decades, increasing numbers of adult patients have sought orthodontic treatment and expressed a desire for esthetic and comfortable alternatives to conventional fixed appliances [10, 11]. Invisalign (Align Technology, Inc., Santa Clara, CA, USA) is an orthodontic system that has been introduced to answer this request. Several case reports [12–14] have shown the possibility of obtaining class II correction with a sequential maxillary molar distalization in non-growing subjects. However, a sound clinical judgment should always be made on the basis of a higher level of evidence.

Simon et al. [15] reported a high accuracy (88 %) of the bodily movement of upper molars with aligners when a mean distalization movement of 2.7 mm was prescribed. The authors reported the best accuracy when the movement was supported by the presence of an attachment on the tooth surface. Furthermore, they underlined the importance of staging in the treatment predictability.

However, a detailed analysis of the underlying skeletal and dental changes induced by aligners during class II treatment in adult patients is still lacking.

On the basis of these considerations, a retrospective multicenter study has been conducted to analyze dentoalveolar and skeletal changes following maxillary molar distalization therapy with the Invisalign protocol in adult patients. The study was conducted in order to test the hypothesis that maxillary molar bodily distalization is not achievable with aligners.

## Methods

### Subjects

A sample of 32 Caucasian subjects treated with distalizing Invisalign aligners was recruited by two board-certified orthodontists. All patients met the following inclusion criteria: (1) age more than 18 years old, (2) skeletal class I or class II malocclusion and a bilateral end-to-end class II molar relationship, (3) normodivergence on the vertical plane (SN^GoGn angle less than 37°), (4) mild crowding in the upper arch (≤4 mm), (5) absence of mesial rotation of the upper first molar according to Ricketts [16], (6) standardized treatment protocol, (7) good compliance during the treatment (wearing aligner time, ≥20 h per day), (8) absence or previous extraction of the upper third molars, and (9) good quality radiographs, with adequate landmark visualization and head rotation control.

The exclusion criteria were (1) transversal dental or skeletal discrepancies, (2) vertical dental or skeletal discrepancies, (3) extraction treatment (except for third molars), (4) unilateral distalization, (5) signs and/or symptoms of temporomandibular disorders (TMDs) accordingly to Diagnostic Criteria for TMDs [17], (6) periodontal disease, (7) endodontic treatments of the maxillary molars, (8) prosthodontics rehabilitations of the maxillary molars.

To avoid selection bias, all subjects who met the inclusion criteria were included in the study regardless the treatment results.

From the initial sample, 12 subjects were excluded according to defined criteria: poor film quality or incomplete records (2 patients), divergence measured at SN^GoGn angle more than 37° (6 patients), unilateral distalization (3 patients), and prosthesis on first molars (1 patient). The final sample consisted of 20 Caucasian adult patients (9 males, 11 females; mean age and SD 29.73 ± 6.89)

Forty lateral cephalograms in habitual occlusion were thus considered for the study. Cephalometric headfilms were collected at the beginning (T0) and at the end of the Invisalign orthodontic treatment (T1). The mean time period between the initial T0 radiograph and the post-treatment T1 radiograph was 24.3 ± 4.2 months. Gender differences were not considered since only non-growing subjects were considered for the study.

All the patients were treated with the Invisalign appliance by two board-certified orthodontists in orthodontic private practices located in Torino (Italy) and Vancouver (Canada). The standardized orthodontic intervention was represented by the maxillary molar distalization protocol proposed by Align Technology: the ClinCheck® (software developed by Align Technology in order to provide the doctor a virtual 3-D simulation of the planned orthodontic treatment based on the patient’s beginning situation and the doctor’s predescribed treatment plan) of each treated case was planned in order to obtain a sequential distalization on the upper arch, and the staging was set at 0.25 mm per aligner. Sequential distalization simply means that the aligners are set up to distalize one tooth at a time (as opposed to en masse movements) The distalization starts with the upper second molars, and once the second molars are two thirds of the way, then the upper first molars move back, then premolars, and so on until the en masse retraction of the four incisors will complete the treatment plan [18]. The protocol comprises the use of attachments and class II elastics. Intermaxillary elastics were used during the retraction of premolars, canines, and incisors. The attachments were engineered by Align Technology to achieve predictable tooth movements and placed according to the Align Technology attachment protocol [19]. In order to control the distalization movement, rectangular and vertical attachments were placed on the distalizing teeth of all patients (from canine to second molar) [12]. In a sequential distalization setup, distalization is built into the aligners and it is the aligners that move the teeth back, not the elastics [18]. As the molars are distalized with the aligners, the molars are pitted against the rest of the arch for anchorage. To prevent loss of anchorage and thus possible flaring of the anterior teeth, class II elastics (1/4 in., 4.5 oz Ormco Corp., Glendora, CA, USA) are used to reinforce the anchorage

Patients selected for the study satisfied the compliance criteria of wearing aligners and class II elastics at least 22 h per day as recommended by Align Technology with regular 6-week monitoring for encouragement.

Thus, all the selected patients were treated with this standardized procedure without any other auxiliaries. Interproximal reduction was not applied.

The average number of required aligners was 42.6 ± 4.4 on the upper arch and 21.4 ± 3.2 on the lower arch. Each couple of aligner was worn for 14 days, as recommended by the manufacturer. A refinement phase, corresponding to the finishing phase, with a mean number of 9.1 ± 2.2 aligners on the upper arch and 6.7 ± 3.1 on the lower arch was requested for each case: during the refinement phase, each aligner was worn for 10 days. The mean treatment time was 24.3 ± 4.2 months.

Informed consent was obtained from each subject. The study was conducted in accordance with the Declaration of Helsinki. The study was registered on the ISRCTN register (ISRCTN66553029) and was approved by the local ethics committee (#3732015 Ethics Board of City of Health and Science, Turin).

### Cephalometric analysis

For each patient enrolled in the study, pre- and post-treatment lateral radiographs were collected. Different X-ray devices for cephalometric radiographs were used, and for this reason, lateral cephalograms for each patient at T1 and T2 were standardized to life size using the ruler present in each X-ray examination [20]. The digital X-rays were stored in a computer and imported into a commercial software (OriSCeph Rx3, Elite Computer, Vimodrone, Italy), in order to perform landmark identifications and cephalometric tracings. These operations were randomly performed by one investigator blinded about the study (SR), using a customized digitization set including 42 landmarks and 39 variables chosen from different cephalometric analyses [21–26]. The large number of variables was due to the number of analyzed teeth and the number of analyzed crown and root landmarks.

All the cephalograms were traced again after 3 weeks and then after 6 months. If there was a discrepancy between the three cephalograms, a new tracing was obtained by mutual agreement (SR, AD, TC).

On the lateral headfilms, the palatal plane/mandibular plane (PP/MP) and the SN/mandibular plane angles were evaluated as indicators of skeletal posterior vertical dimension changes [27].

**Fig. 1:**
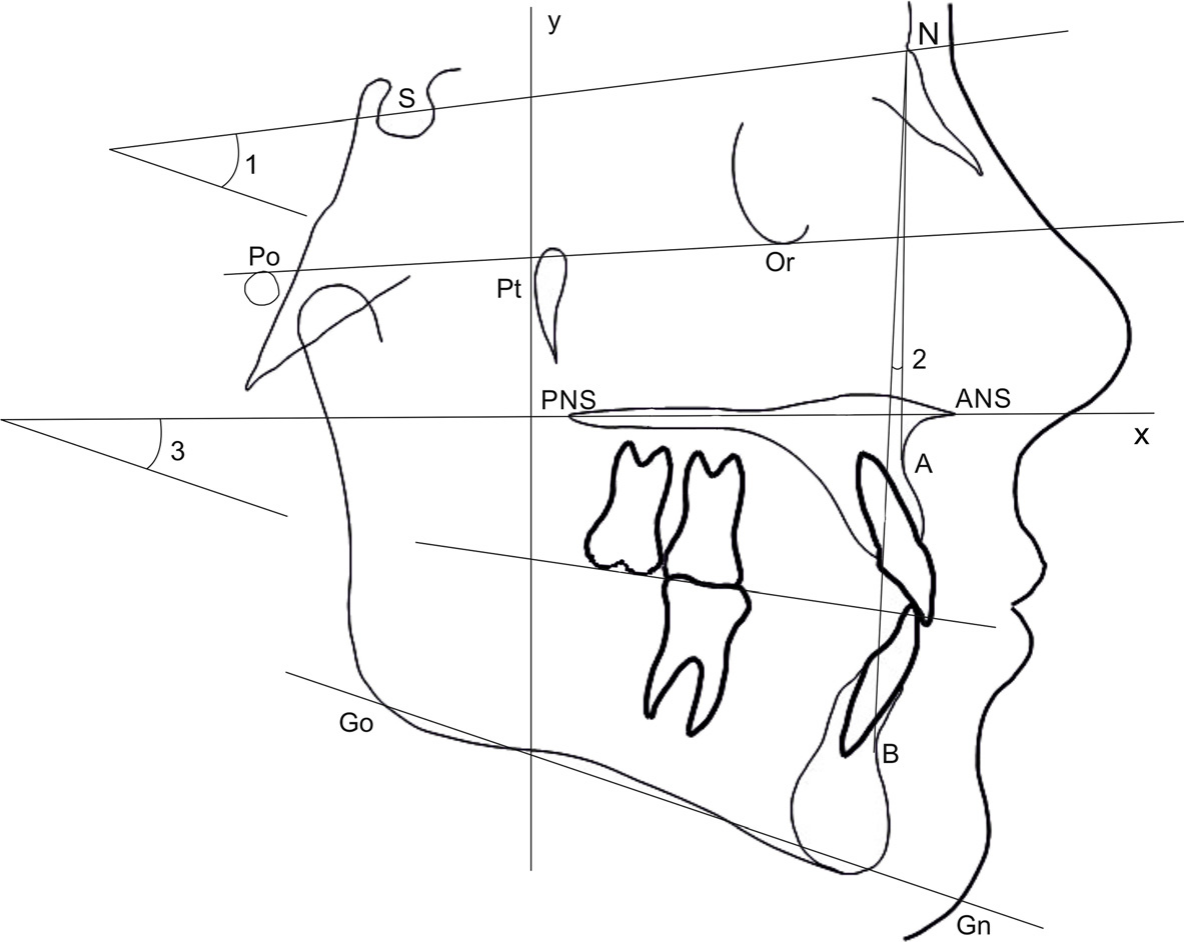
Schematic illustration of the skeletal variables considered in the study. *1* SN^GoGn (°); *2* SN^A (°), SN^B(°), AN^B (°); *3* SpP^GoGn (°)

**Fig. 2:**
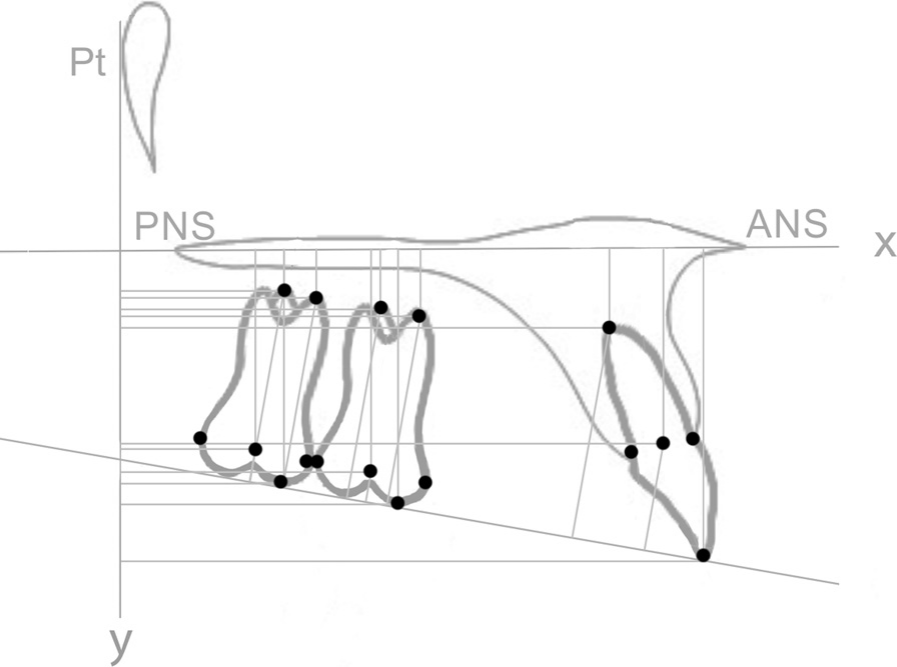
Schematic Illustration of angular measurements (°) of maxillary second molar (1), maxillary first molar (2), and central incisor (3). The angle between the tooth long axis (passing trough the mesiobuccal cusp and mesiobuccal root’s apex for the first and the second molar; passing trough the incisal edge and root’s apex for the central incisor) and *x* axis (palatal plane) expressed the inclination of the tooth

**Fig. 3:**
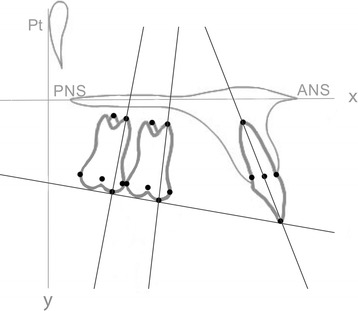
Schematic illustration of linear measurements (mm) considered in the study. Horizontal measurements were expressed by the distance between the following points and the y axis (a perpendicular line to the palatal plane passing through the Ricketts’ Pt point); second molar horizontal distance from the center of the crown, from the mesiobuccal cusp, from the mesiobuccal root’s apex, from the palatal root’s apex; first molar horizontal distance from the center of the crown, from the mesiobuccal cusp, from the mesiobuccal root’s apex, from the palatal root’s apex; central incisor distance; central incisor edge distance, central incisor radicular apex distance. Vertical distances were expressed by the distance between the same points and the x axis (palatal plane) and between the occlusal plane (except for incisor edge point and mesiobuccal cusp point, both tangential the occlusal plane)

On the initial (T0) and final (T1) cephalograms, the reference axes were represented by the palatal plane (*x* axis) and by a perpendicular line to the palatal plane passing through the Ricketts’ Pt point (*y* axis) (Fig. 1). The occlusal plane was traced as well, passing trough the upper central incisor’s incisal edge and the mesial cusp of the first molar [24]. The palatal plane was used to measure vertical and angular movements (Fig. 2), the occlusal plane was used to measure vertical movements only, while the *y* axis was used to measure sagittal movements of the second molar, of the first molar, and of the central incisor (Fig. 3).

Crowns’ centers, obtained as the midpoint between the greatest mesial and distal convexity of the crown, as well as the axis passing through mesial cusps and mesiovestibular roots’apex were taken as reference points of the maxillary first and second molar as seen on the cephalograms [22]. The reference point for the central incisor was the midpoint of the lateral projection of the circumference formed by the root and crown conjunction [24].

The overall craniofacial treatment changes were evaluated by superimposing on the stable structures of the anterior cranial base according to the structural method [28]. Superimpositions were conducted digitally.

Regional superimpositions in the maxilla were made along the palatal plane (internal structures of maxilla) and the constructed *y* axis not to evaluate changes in the maxilla, since no significant differences occur in the palatal plane in non-growing patients [23], but to assess the amount of molar distalization [26]. In case of left- and right-side cephalometric superimposition, the landmark considered both at T0 and T1 was the more distal positioned.

The considered landmarks and maxillary measurements are described in Figs. 1, 2, and 3.

### Statistical analysis

Statistical analysis was performed using the R statistical package (version 3.0.1, R Core Team, Foundation for Statistical Computing, Vienna, Austria). The normality assumption of the data was evaluated with the Shapiro-Wilk test. According to this evaluation, the differences between before (T0) and after treatment (T1) were compared with the *t* test. The level of significance was set at *P* < 0.05.

Reproducibility of measurements was assessed by the intraclass correlation coefficient (ICC). ICC provides the percentage of the total variance of the measures that can be attributed to the variability between subjects. The remaining percentage of variance is due to repeated trials. ICC values higher than 80 % indicate excellent reproducibility, whereas values below 60 % reflect poor reproducibility. ICC between 60 and 80 % is considered good reproducibility [29].

## Results

The mean, standard deviation, and 95 % CI values of the change in dental and skeletal variables are reported in Table 1. Significant changes in the sagittal positions of upper first and second molars (*P* < 0.01) were revealed after distalization. The second molar showed a distal average movement of 2.52 mm measured on the mesiobuccal cusp and of 2.12 mm measured on the center of the crown, without significant tipping (*P* = 0.056) and vertical movements of the crown (*P* = 0.25). The mean amount of maxillary first molar distalization was 2.25 mm measured on the mesiobuccal cusp and 2.03 mm on the center of the crown, without significant vertical movements of the crown (*P* = 0.43) and tipping movements (*P* = 0.27).

**Table 1:** Study group: difference of means between T0 and T1 for all the considered variables

Variable	T0		T1		Difference of means	95 % CI		Significance
	Mean	SD	Mean	SD	(T1–T0)	Lower	Upper	*P* value
SN^GoGn	32.80	7.16	32.35	6.43	−0.45	−1.20	0.30	N.S.
SNA	81.00	4.08	80.70	4.44	−0.30	−1.12	0.52	N.S.
SNB	76.45	2.76	76.95	3.45	0.50	−0.19	1.19	N.S.
ANB	4.45	2.91	3.75	2.95	−0.7	−1.29	−0.11	0.023*
SPP^GoGn	26.10	6.08	26.20	5.75	0.10	−1.05	1.25	N.S.
17mcPtV	14.99	3.41	12.47	2.84	−2.52	−3.24	−1.79	0.0000006517****
17ccPtV	13.87	3.09	11.75	2.69	−2.12	−2.76	−1.48	0.000001402****
17praPtV	16.25	2.58	14.74	2.31	−1.50	−2.07	−0.94	0.00002191****
17vmraPtV	18.24	2.67	16.57	2.48	−1.67	−2.31	−1.03	0.00003057****
16mcPtV	24.91	3.93	22.65	3.97	−2.25	−4.21	−0.29	0.027*
16ccPtV	23.17	3.80	21.14	3.24	−2.03	−2.72	−1.35	0.000005878****
16praPtV	24.39	3.33	22.54	2.58	−1.84	−2.86	−0.82	0.001***
16vmraPtV	26.66	3.27	25.17	3.02	−1.48	−2.40	−0.57	0.003**
11raPtV	45.14	4.58	44.34	4.15	−0.81	−2.35	0.73	N.S.
11ccPtV	49.86	5.13	48.54	5.11	−1.31	−2.83	0.21	N.S.
11iePtV	53.50	5.82	51.28	5.87	−2.23	−3.76	−0.70	0.007**
17mcPP	20.39	3.19	19.91	2.43	−0.49	−1.59	0.62	N.S.
17ccPP	16.66	2.83	16.15	2.22	−0.51	−1.40	0.39	N.S.
17praPP	4.86	3.55	3.58	1.98	−1.28	−3.09	0.53	N.S.
17vmraPP	3.70	1.82	3.59	2.20	−0.11	−0.90	0.68	N.S.
16mcPP	23.15	2.68	22.53	2.59	−0.62	−1.44	0.19	N.S.
16ccPP	19.36	2.39	19.06	2.32	−0.31	−1.11	0.49	N.S.
16praPP	6.72	1.60	6.17	2.01	−0.55	−1.45	0.34	N.S.
16vmraPP	5.53	1.93	4.73	2.10	−0.80	−1.67	0.06	N.S.
11raPP	5.64	2.50	5.00	2.34	−0.63	−1.69	0.43	N.S.
11iePP	28.98	2.98	28.62	2.85	−0.36	−1.28	0.57	N.S.
11ccPP	20.39	7.95	19.24	2.49	−1.15	−4.59	2.29	N.S.
17mcOP	1.28	0.99	0.99	0.89	−0.29	−0.80	0.23	N.S.
17ccOP	4.06	1.74	4.07	1.46	0.01	−0.72	0.74	N.S.
17praOP	16.82	2.29	16.95	1.81	0.13	−0.82	1.09	N.S.
17vmraOP	16.97	3.70	17.42	1.96	0.44	−1.27	2.25	N.S.
16ccOP	3.21	0.96	3.16	0.78	−0.05	−0.55	0.46	N.S.
16praOP	15.86	2.06	16.10	2.11	0.24	−0.43	0.90	N.S.
16vmraOP	17.50	1.93	17.97	2.10	0.48	−0.41	1.36	N.S.
11raOP	21.66	2.77	21.72	2.93	0.06	−1.41	1.54	N.S.
11ccOP	9.20	1.47	8.63	1.34	−0.57	−1.33	0.20	N.S.
17^PP	79.17	6.84	76.53	5.13	−2.64	−5.37	0.06	N.S.
16^PP	84.64	7.82	83.00	4.54	−1.64	−4.67	1.39	N.S.
11^PP	109.60	6.70	106.70	6.66	−2.87	−5.06	−0.69	0.013*

*P < 0.05; **P < 0.01; ***P < 0.001; ****P < 0.0001

For each variable, there is a number indicating the FDA code for the considered tooth; then in small letters, the anatomic point considered (*mc* mesial cusp, *cc* center of the crown, *pra* palatal root apex, *vmra* vestibulo-mesial root apex, *ie* incisal edge, *ra* root apex); then in capital letters, the plane to which the distance from that point is measured (*PtV* line passing trough the Pt Ricketts point, perpendicular to palatal plane; *PP* palatal plane; *OP* occlusal plane). The italic values highlight the significance of the amount of the distalization movement. *N.S.* Not Significant

**Fig. 4:**
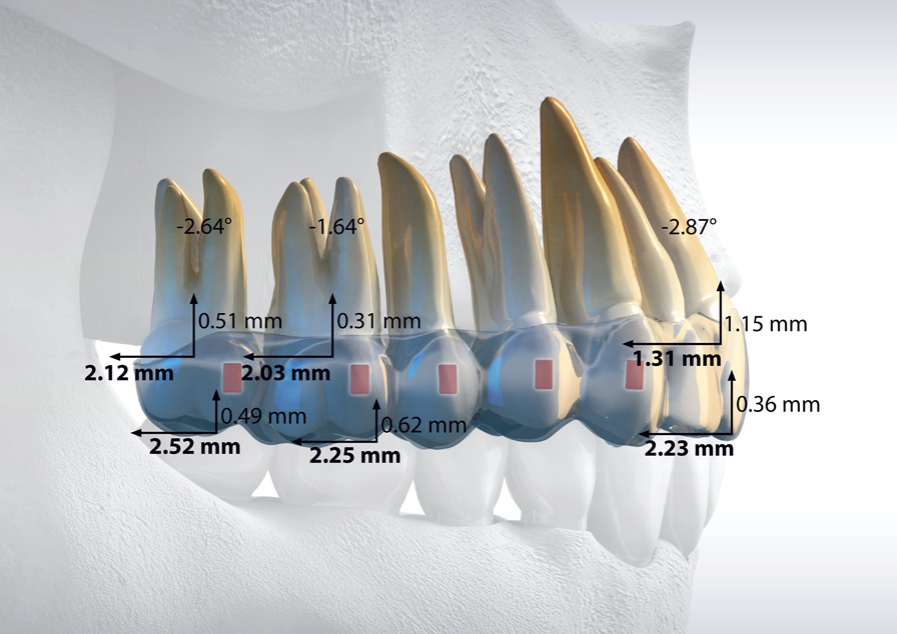
Schematic illustration of clinically relevant results for the study

The maxillary central incisor edge was retracted by 2.23 mm (*P* < 0.01) without significant vertical movements (*P* = 0.43) and with a good control of its orientation with respect to the palatal plane (initial value 109.60° ± 6.70°, post-treatment value 106.70° ± 6.66°, *P* < 0.05) (Fig. 4).

With regard to skeletal changes of the maxilla, the SN^A (angle measured at Sella point, Nasion point, A Downs point) angle showed no statistical differences between pre- and post-treatment cephalograms (*P* = 0.45) (Table 1). The craniofacial vertical dimension was not affected by the distalization of maxillary molars with aligners. SN^GoGn and SPP^GoGn angles showed no significant differences between pre- and post-treatment cephalograms (*P* = 0.22 and *P* = 0.85, respectively).

The ICC showed excellent reproducibility for linear measurements (96 %) and angular measurements (94 %).

## Discussion

The aim of this study was to evaluate the Invisalign aligner’s effects in distalizing maxillary molars. Results indicate the possibility of a translation movement of maxillary molars at least when a minimal correction on the sagittal plane is required. Thus, the hypothesis of the study was rejected. A recent work by Simon et al. [30], in an in vitro setup, demonstrated that forces and moments generated by Invisalign aligners for the distalization movement are consistent with the orthodontic literature values. In particular, the initial mean forces in the direction of the movement were about 1.0 N when an attachment was associated. The amount of distal movement of the maxillary molars obtained in this study (first molar average movement 2.25 mm, second molar average movement 2.52 mm) was comparable to those obtained on a sample of ten patients by Simon et al. (average movement 2.6 mm) even if they measured the movement only on the horizontal plan, without considering vertical and angular movements accompanying the distalization. In the present study, an evaluation of the molars’ tipping and vertical movements while distalizing was performed. To our knowledge, this is the first study in which this evaluation has been performed.

In another work by Simon et al. [15], the molar distalization with aligners revealed an accuracy of 87 % confirming a good performance of the appliance when a maximum distalization of 3 mm was requested. Results from the present study and from the studies by Simon et al. [15, 30] are in contrast with results obtained by Djeu et al. [31], which reported that Invisalign was especially deficient in treating anteroposterior discrepancies. However, in their study, Djeu et al. compared adult patients treated with aligners with younger patients, comprising pre-adolescent patients; they did not report the staging of the aligners and the distalization protocol used. Furthermore, information regarding the use of attachments were not provided. Thus, their results should be considered with extreme caution.

**Fig. 5:**
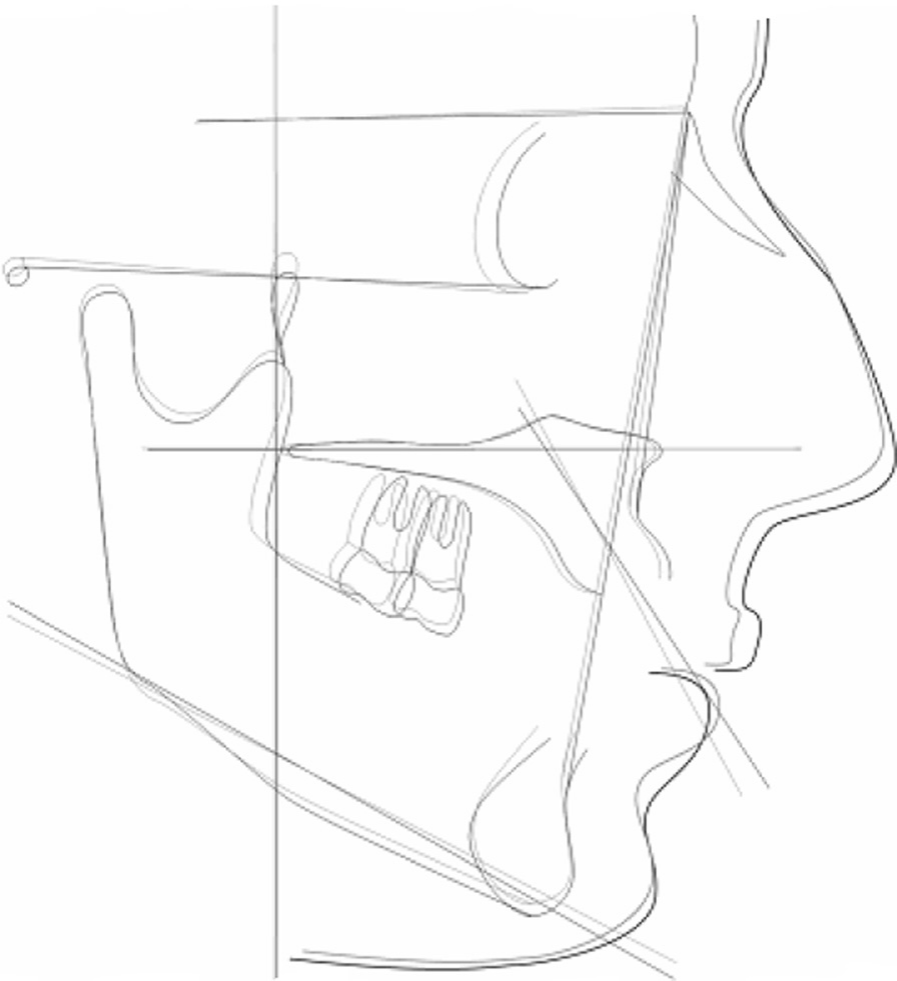
Superimposition of one of the patients treated in this study

**Fig. 6:**
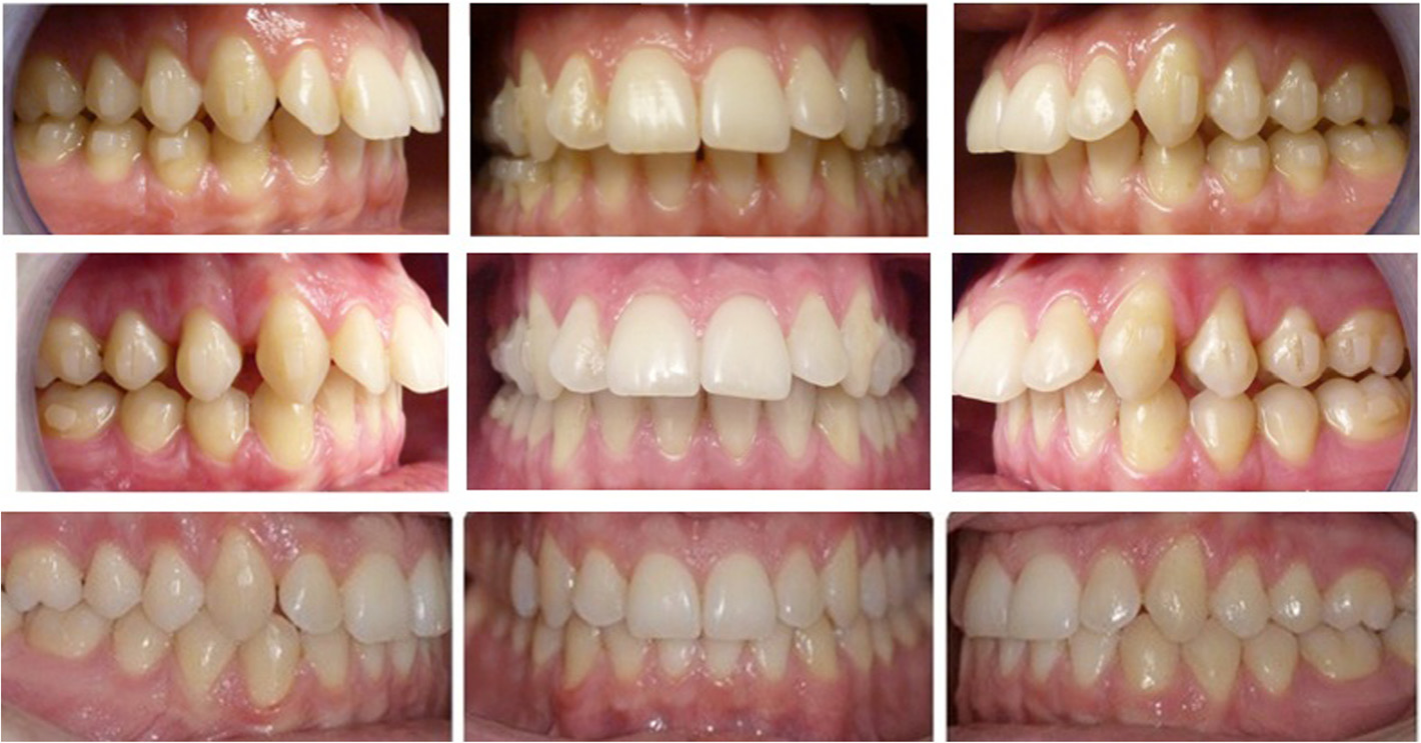
Pre- and post-photos of one of the patients treated in this study

In an adult patient, class II correction comes primarily from tooth movement without the benefits of growth and molar distalization could be usually performed to gain 2 to 3 mm of space in the dental arch to achieve a class I relationship [32]. In order to obtain this amount of movement, upper third molars, if present, should be extracted to have enough “room” to move second and first molars in end-to-end class II malocclusions (Figs. 5 and 6). Furthermore, considering that the correction comes primarily from tooth movement, more anchorage control is required [33].

When reviewing several studies conducted on intraoral non-compliance appliances, dentoskeletal effects revealed anchorage loss at the reactive part, distal tipping, and extrusion of molars [2–4, 22–26]. Usually, the anchorage loss occurred particularly in the incisor area due to the reciprocal force reacting to the distalizing force. Early observations [13] confirmed that the use of class II elastics during maxillary molar distalization with aligners prevents the uncontrolled proclination of the anterior teeth. Furthermore, the sequential distalization protocol limits space opening between the distalizing teeth which is not only more esthetic but maintains maximum aligner contact with the teeth and reduces the flexibility of the plastic material. That, in turn, minimizes uncontrolled incisor tipping, which is expressed clinically as increased overbite with a loss of palatal root torque [34]. As a result of this clinical approach, in our study, upper incisors were retroclined by 2.87° and retracted by 2.23 mm with a good control of their buccolingual inclination with respect to the palatal plane. However, this evaluation was performed at the end of the treatment and not at the end of the distalizing phase; thus, a final conclusion cannot be drawn.

The distalization movement with Invisalign aligners was not associated with significant distal tipping of the distalized molars. The self-limiting 0.25-mm activation of each aligner (as opposed to the more continuous activation of nickel titanium springs or elastomeric chain) means that any tip created by the aligner during space closure is probably not due to the teeth “falling” or even being “pushed” into a pontic space, or to a lack of countermoment surface, but to insufficient moments being generated to control root movement [34]. Rectangular and vertical attachments located on the buccal aspect of the distalizing molars are required in order to create a sufficient moment to oppose the tipping movement [35].

The tipping standard deviations (5.13° for the second molar and 4.54° for the first molar) were indicative of a wide variability. This might be due to individual morphological variations of the maxillary sinus and the alveolar arch, which might have affected the amount of distalization [36].

The patient’s vertical growth pattern is an important point to consider while planning molar distalization. A clockwise rotation of the mandible due to premature contacts may worsen the profile and cause bite opening. The distal movement measured in our study was not associated with extrusion or intrusion movements of the teeth. However, the thickness of the aligners and the consequent bite block effect might explain the absence of any change of anterior vertical dimension [37].

Class II elastics seemed to have any effect on the lower arch: any significant tipping of the lower first molar or proclination of the lower incisors was revealed.

Although these results are encouraging, this topic would need further investigation, for example, with randomized clinical trials and a larger sample size. Retrospective studies have some disadvantages with respect to prospective studies. Among the biases which can negatively impact the veracity of this type of study are selection bias and misclassification or information bias as a result of the retrospective aspect. However, it is quite difficult to conduct a prospective study investigating the effects of an uncommon clinical procedure due to the difficulties to achieve a proper sample size. This is the reason why private practices were involved, and this is the reason why the retrospective design seemed to be the more indicated study design at this stage of our knowledge on aligner orthodontics. To avoid selection bias, all subjects who met the inclusion criteria were included in the study regardless the treatment result. However, we are aware that a prospective study could lead to less significant results considering a proper sample selection and the risk of dropouts of the study design.

Other limitations of the present study were represented by the lack of a separate evaluation of the treatment effects of aligners and class II elastics and the lack of compliance monitoring.

Class II elastics were used after the distalization of the second and first molars; thus, the molar distalizing movements were related only to the aligner’s effect, while the measured effects on the incisors should be evaluated with caution since incisor retrusion was obtained with aligners and class II elastics.

Attempts were made to monitor the compliance asking each patient to fulfill a monthly diary; however, most of the patients failed to report complete monitoring during the treatment. The insertion of compliance indicators based on the food dye Erioglaucine disodium salt, which is encapsulated in the Invisalign Teen**®** aligners and released from the polymer in the presence of oral fluid, in every Invisalign aligner could be very helpful to monitor the treatment of adult patients too [38]. However, considering that those compliance indicators are not immune from intentional or unintentional manipulations [39], more objective compliance evaluation tools are recommended. Further researches are warranted to evaluate class II elastics effects and patient compliance with different compliance indicators and their impact on tooth movement.

Dentoskeletal effects of class II correction with aligners has never been described before: most of the published papers are case reports [12–14, 22] including both growing and non-growing subjects.

In a recent review, Rossini et al. [40] stated that Invisalign aligners are effective in controlling upper molar bodily movement when a distalization of 1.5 mm has been prescribed. Our results confirmed that Invisalign aligners proved to be suitable for distalizing maxillary molars when a distalization of 2 to 3 mm is required to obtain a class I relationship in selected end-to-end class II adult patients.

## Conclusions

Within the limitations of a retrospective study design, and of a small sample size, this study demonstrated that Invisalign aligners are effective in distalizing maxillary molars in non-growing subjects without significant vertical and mesiodistal tipping movements. As a consequence, the lower facial height did not change at the end of the treatment. Therefore, clinicians can consider the use of Invisalign aligners in treatment planning for adult patients requiring 2 to 3 mm of maxillary molar distalization.
